# Impact of non-amyloid cerebral small vessel disease on the efficacy and safety of argatroban in acute ischemic stroke: a retrospective cohort study

**DOI:** 10.3389/fneur.2026.1861639

**Published:** 2026-08-11

**Authors:** Fen Liu, Qiang Wu, Yan Lin, Jie Chen

**Affiliations:** 1 Department of Neurology, Shengli Clinical Medical College of Fujian Medical University, Fuzhou University Affiliated Provincial Hospital, Fujian Provincial Hospital, Fuzhou, Fujian Province, China; 2 Second Department of Internal Medicine, Songxi County Hospital, Songxi, Fujian, China

**Keywords:** acute ischemic stroke, argatroban, cerebral small vessel disease, effect modification, white matter hyperintensities

## Abstract

**Objective:**

To investigate whether imaging features of non-cerebral amyloid angiopathy cerebral small vessel disease (non-amyloid CSVD) influence the efficacy of argatroban in acute ischaemic stroke (AIS) and to test for effect modification.

**Methods:**

Patients with AIS admitted to Songxi County Hospital from January 2022 to December 2023 were enrolled and divided into an argatroban group and a control group. CSVD imaging markers (multiple lacunar infarcts, multiple subcortical white matter lesions, white matter hyperintensities, and brain atrophy) were assessed. Multivariable logistic regression was used to analyse favourable outcome at 90 days (modified Rankin Scale score 0–2), and interaction terms were introduced to test for effect modification.

**Results:**

A total of 139 patients were included (66 in the argatroban group, 73 in the control group). Argatroban reduced early neurological deterioration (adjusted OR 0.045, *p* = 0.004), but no significant difference was observed in the rate of favourable outcome at 90 days between the two groups (80.3% vs. 79.5%, *p* = 0.208). Severe white matter hyperintensities modified the efficacy of argatroban (*p* for interaction < 0.10), with greater benefit seen in patients without severe white matter hyperintensities. Only one intracranial haemorrhage occurred, and it was in the control group.

**Conclusion:**

In patients with AIS after exclusion of cerebral amyloid angiopathy, argatroban improves early neurological function but does not improve functional outcome at 90 days. Severe white matter hyperintensities may be potential effect modifiers, generating hypotheses for imaging-based treatment stratification that warrant prospective validation.

## 1 Introduction

**Figure 1:**
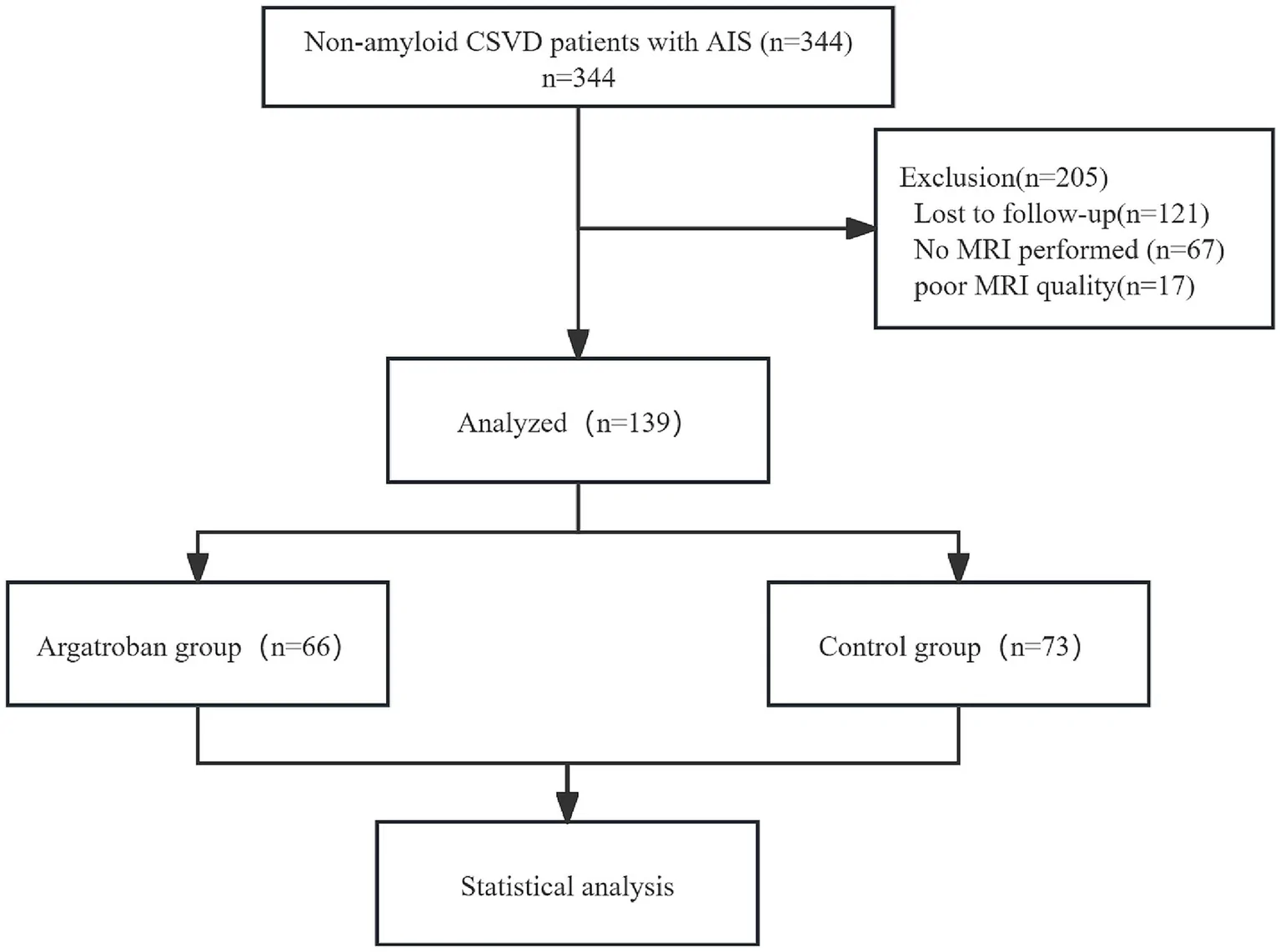
Study flow diagram. A total of 344 patients with non-amyloid CSVD-related acute ischemic stroke (AIS) were assessed. After excluding 205 patients due to loss to follow-up (*n* = 121), no MRI performed (*n* = 67), or poor MRI quality (*n* = 17), 139 patients were included in the final analysis. Among them, 66 patients received argatroban plus standard antiplatelet therapy (argatroban group) and 73 patients received standard antiplatelet therapy alone (control group). All patients completed the 90-day modified Rankin Scale (mRS) assessment and were included in the statistical analysis.

Cerebral small vessel disease (CSVD) is a major cause of cognitive impairment and vascular dementia in the elderly (1). Its imaging features include lacunes, white matter hyperintensities, and microbleeds (2). CSVD is classified into non-amyloid CSVD (hypertension-related) and cerebral amyloid angiopathy (CAA) (3, 4). Patients with CSVD who experience acute ischaemic stroke (AIS) present with more severe neurological deficits, worse functional outcomes, and a higher risk of bleeding after antithrombotic therapy (5–7) (Figure 1).

**Figure 2:**
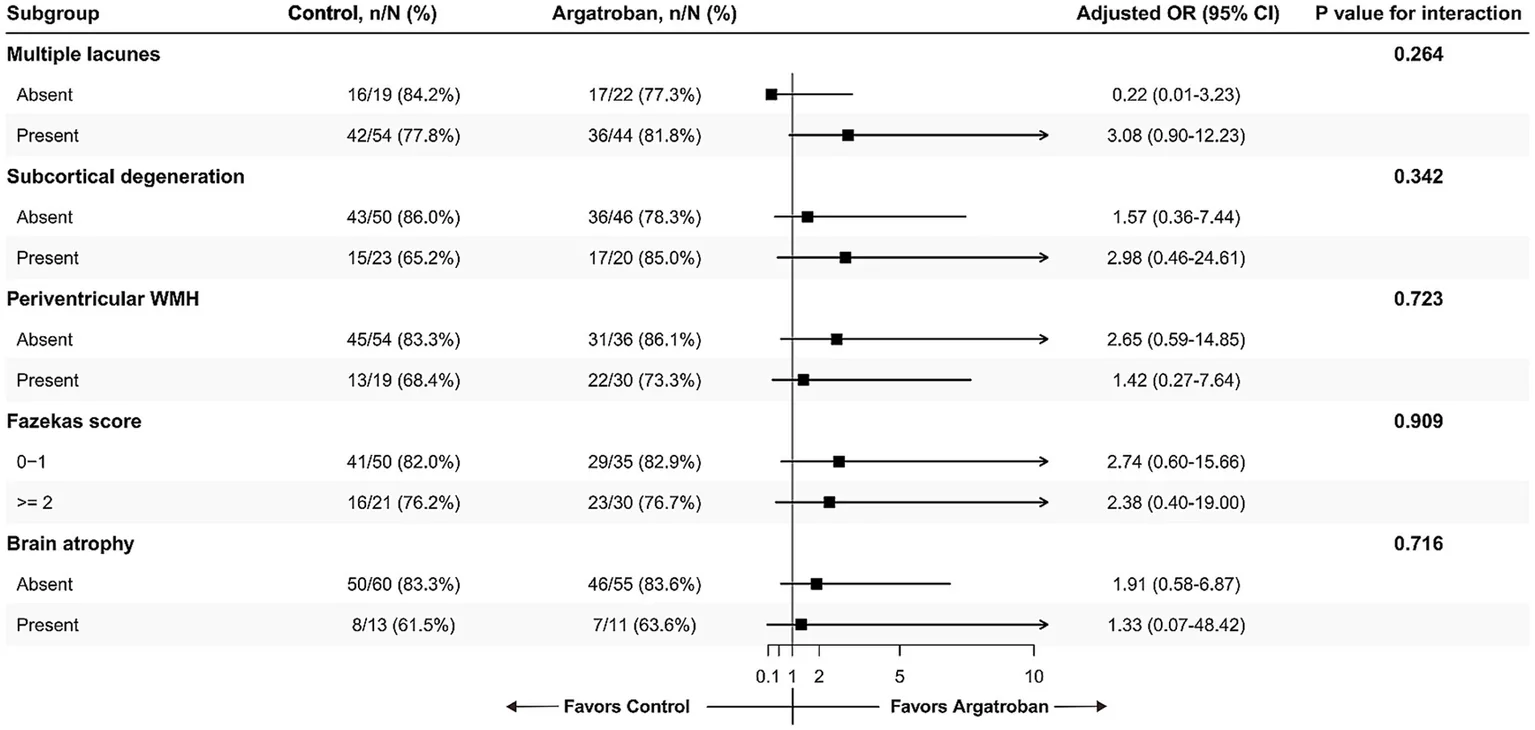
Forest plot of subgroup analyses for the effect of argatroban on 90-day favourable outcome (modified Rankin Scale score 0–2). Odds ratios (ORs) and 95% confidence intervals (CIs) are shown for each subgroup, stratified by imaging features of cerebral small vessel disease. The size of each square is proportional to the weight of the subgroup in the analysis. Horizontal lines represent 95% CIs. The vertical dashed line indicates the null effect (OR = 1). *p* values for interaction are displayed on the left. NE, not estimable due to zero events in the argatroban subgroup.

Argatroban is a direct thrombin inhibitor that exerts rapid anticoagulant effects (8) and has been approved for the acute phase of AIS in countries including Japan and China (9, 10). The ARGIS-1 and ARTSS-2 trials have demonstrated its safety and early benefit (11, 12). Although argatroban shows early benefit in the acute phase, whether it improves functional outcome remains uncertain, and the bleeding risk in patients with compromised vessel integrity requires attention (13). Consequently, international guidelines remain conservative regarding its use (14) (Figure 2).

Owing to blood–brain barrier disruption, patients with CSVD may have increased sensitivity to anticoagulation (15). The bleeding risk in CAA is extremely high, making it a contraindication to anticoagulation (16, 17); in contrast, the bleeding risk in non-amyloid CSVD is relatively manageable (18). However, different imaging features may affect treatment efficacy differently: white matter hyperintensities are associated with bleeding risk (19, 20), lacunes with poor recovery (21), and brain atrophy with extensive neuronal damage (22). Treating CSVD as a binary variable (present or absent) may therefore obscure its heterogeneity.

**Figure 3:**
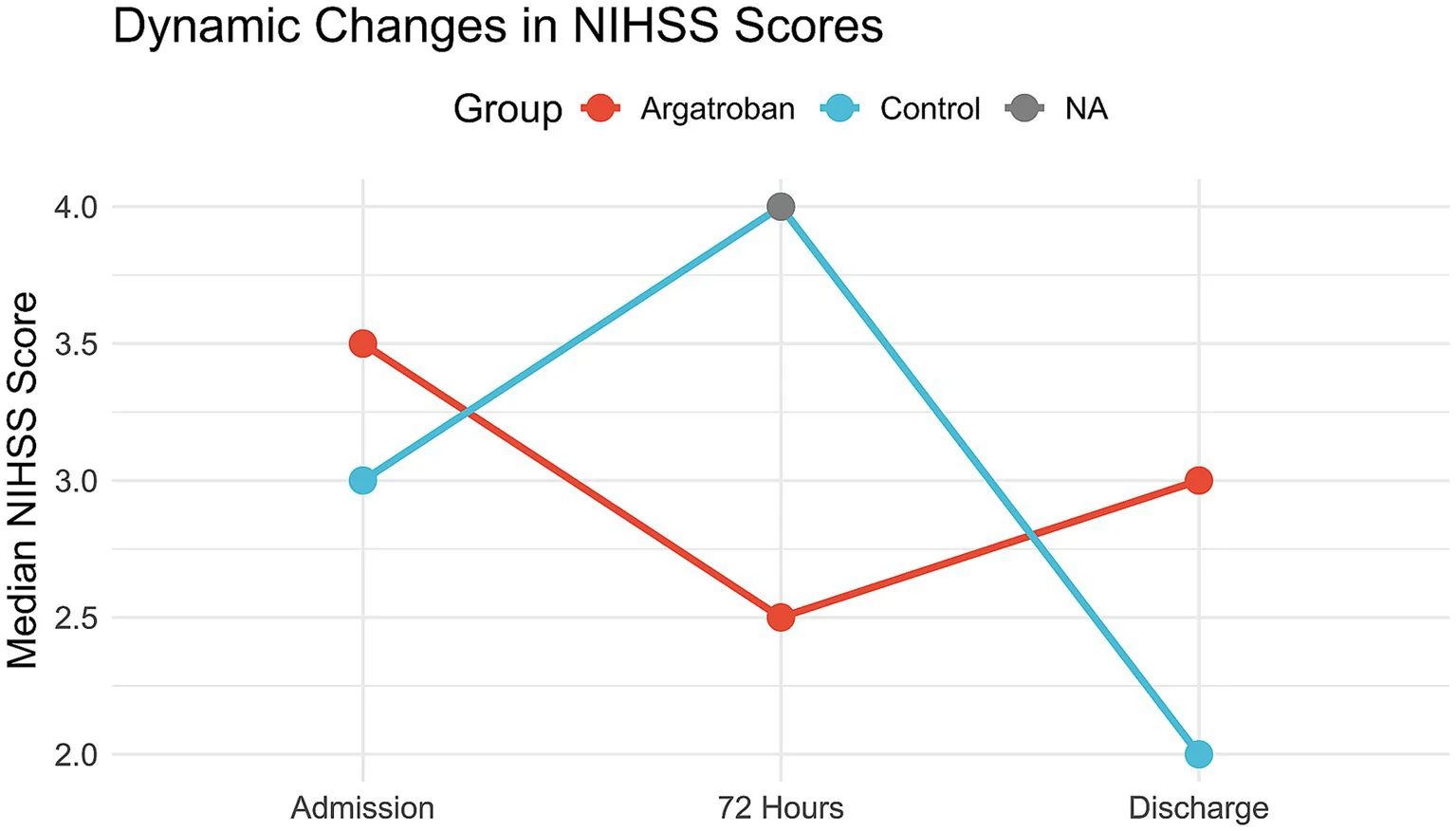
Dynamic changes in National Institutes of Health Stroke Scale (NIHSS) scores from admission to discharge. Median NIHSS scores (with interquartile ranges) are shown for the argatroban group (solid line) and control group (dashed line) at admission, 72 h, and discharge. The argatroban group showed a greater reduction in NIHSS score at 72 h compared with the control group (adjusted *β* = −1.691, *p* < 0.001), but no significant difference was observed at discharge.

Currently, no study has systematically evaluated the impact of imaging features of non-amyloid CSVD on the efficacy of argatroban. This study aims to investigate the influence of four core features: multiple lacunar infarcts, multiple subcortical white matter lesions, severe white matter hyperintensities, and brain atrophy—on the efficacy of argatroban and to test for effect modification through interaction analysis (Figure 3).

## 2 Materials and methods

### 2.1 Study design and participants

**Figure 4:**
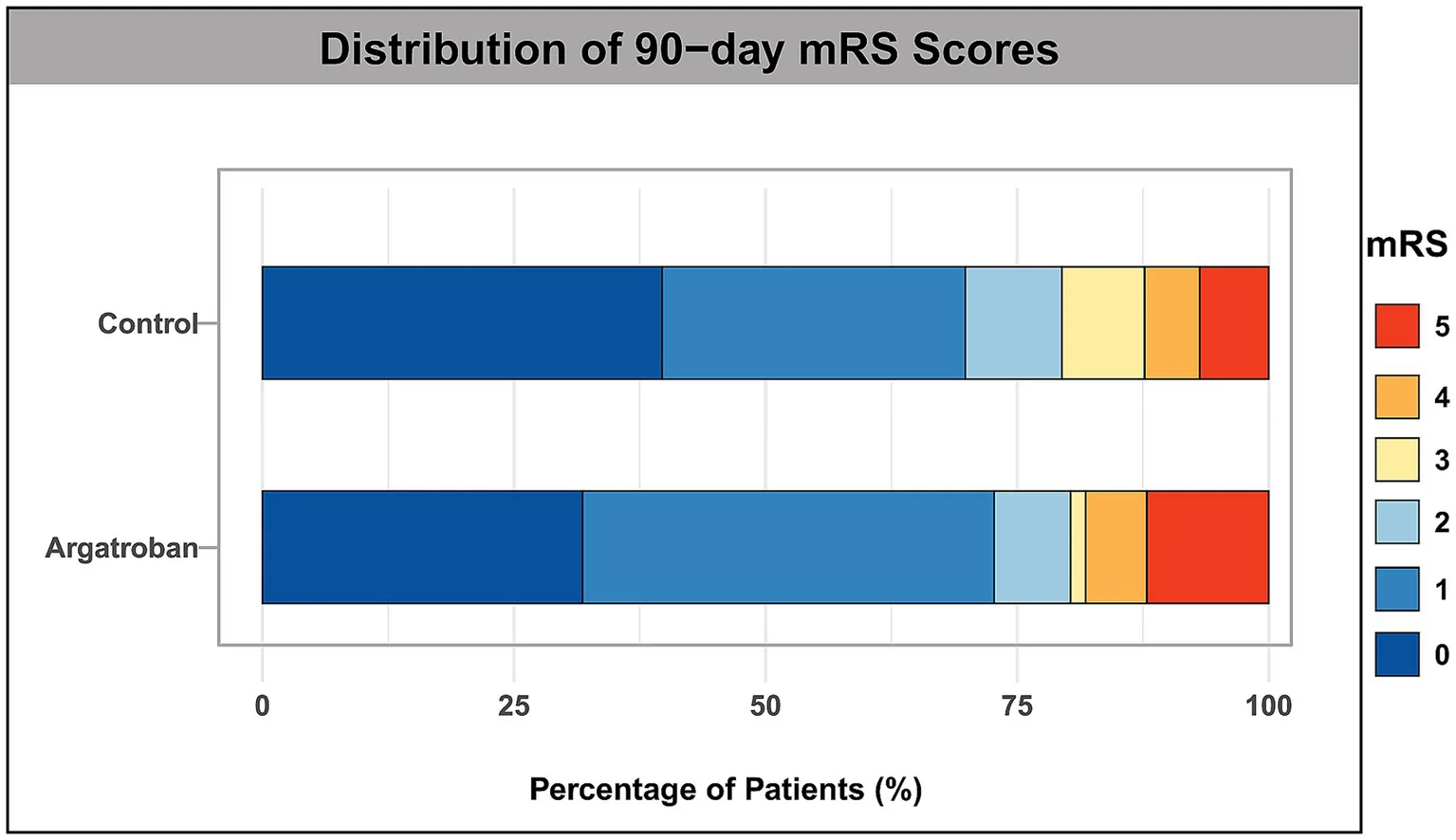
Distribution of 90-day modified Rankin Scale (mRS) scores in the control and argatroban groups. The stacked bar chart shows the proportion of patients achieving each mRS score from 0 to 6. Favourable outcome (mRS 0–2) was achieved by 79.5% of patients in the control group and 80.3% in the argatroban group (adjusted OR 1.899, 95% CI 0.714–5.350, *p* = 0.208). The distribution of mRS scores did not differ significantly between the two groups (*p* = 0.573).

This was a single-centre retrospective cohort study. Patients with AIS admitted to Songxi County Hospital from January 2022 to December 2023 were enrolled. Inclusion criteria were: (1) age ≥ 18 years; (2) admission within 72 h of symptom onset; (3) diagnosis of AIS according to WHO/AHA/ASA criteria (14); (4) treatment with argatroban, standard antithrombotic therapy, or both during hospitalisation. Exclusion criteria were: (1) imaging findings suggestive of probable or possible CAA according to the modified Boston criteria (i.e., presence of ≥2 strictly lobar cerebral microbleeds, cortical superficial siderosis, or spontaneous intracerebral haemorrhage); (2) history of intracranial haemorrhage; (3) severe hepatic or renal dysfunction; (4) incomplete clinical data or MRI of insufficient quality for CSVD assessment (Figure 4).

### 2.2 Grouping and treatment

Patients were divided into two groups based on whether they received argatroban during hospitalisation.

**Figure 5:**
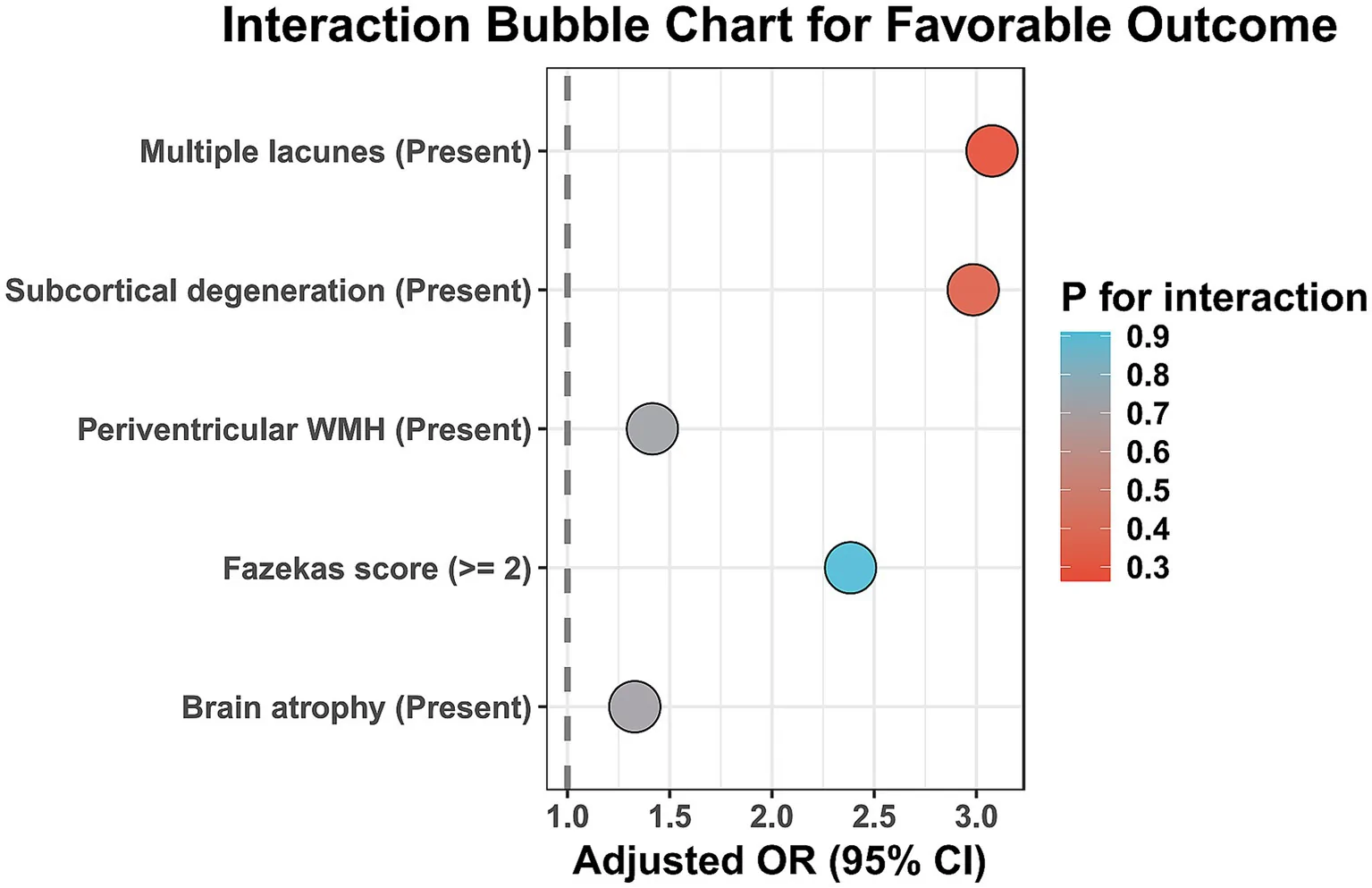
Interaction bubble chart for the effect of argatroban on 90-day favourable outcome across different cerebral small vessel disease (CSVD) imaging features. Each bubble represents a subgroup defined by the presence or absence of a specific CSVD marker. The *x*-axis indicates the odds ratio (OR) for favourable outcome in the argatroban group relative to the control group; the *y*-axis lists the CSVD markers. Bubble size is proportional to the sample size of the subgroup. The vertical dashed line indicates the null effect (OR = 1). Severe white matter hyperintensities (Fazekas grade ≥2 or total score ≥3) showed a significant modifying effect (*p* for interaction < 0.10), with greater benefit in patients without severe white matter hyperintensities (OR 2.740, 95% CI 0.595–15.655) than in those with severe white matter hyperintensities (OR 2.384, 95% CI 0.395–18.999).

Argatroban group: Argatroban injection (Sichuan Kelun, 2 mL:10 mg) was added to standard antiplatelet therapy (aspirin or clopidogrel). The regimen consisted of an initial dose of 60 mg/day by continuous intravenous infusion for 48 h, followed by 10 mg twice daily as an intravenous infusion for 7–14 days (Figure 5).

Control group: Standard antiplatelet therapy alone.

### 2.3 Data collection

Demographic characteristics, vascular risk factors, baseline clinical data (blood pressure, National Institutes of Health Stroke Scale [NIHSS] score), laboratory parameters, and imaging data (infarct volume measured by the ABC/2 method) were collected from the electronic medical record system.

### 2.4 Imaging and CSVD assessment

All patients underwent 1.5 T brain MRI (including T1-weighted, T2-weighted, axial fluid-attenuated inversion recovery [FLAIR], T2*-weighted gradient-recalled echo [GRE] or susceptibility-weighted imaging [SWI], and diffusion-weighted imaging [DWI]) within 72 h of admission.

Two trained neuroradiologists, blinded to clinical information and group assignment, independently evaluated CSVD markers according to the Standards for Reporting Vascular Changes on Neuroimaging (STRIVE) consensus (2) and features of CSVD (3, 4). Disagreements were resolved by a third senior radiologist.

1. Multiple lacunar infarcts: Round or ovoid fluid-filled cavities measuring 3–15 mm in diameter in the subcortical or deep regions, with central hypointensity and peripheral hyperintensity on FLAIR.
2. Severe white matter hyperintensities: Deep Fazekas score ≥ 2 or total Fazekas score ≥ 3 (23).
3. Brain atrophy: Assessed visually (present/absent).
4. Multiple subcortical white matter lesions: Multiple hyperintense foci in the subcortical white matter (present/absent).

Enlarged perivascular spaces were not included due to resolution limitations of 1.5 T MRI.

### 2.5 Outcome measures

1. Primary efficacy outcome: Favourable functional outcome at 90 days, defined as a modified Rankin Scale (mRS) score of 0–2.
2. Secondary efficacy outcomes: Change in NIHSS score at 72 h, NIHSS score at discharge, and change in NIHSS from admission to discharge.
3. Safety outcomes: Symptomatic intracranial haemorrhage, any intracranial haemorrhage, and other bleeding events.

### 2.6 Statistical analysis

Statistical analyses were performed using R version 4.2.0. Continuous variables were tested for normality using the Shapiro–Wilk test. Normally distributed data were expressed as mean ± standard deviation and compared using the independent samples *t*-test; non-normally distributed data were expressed as median (interquartile range) and compared using the Mann–Whitney *U* test. Categorical variables were expressed as frequencies (percentages) and compared using the chi-square test or Fisher’s exact test. All tests were two-tailed, and a *p* value < 0.05 was considered statistically significant. No adjustment for multiple comparisons was made.

Multivariable logistic regression was used to analyse poor functional outcome at 90 days (mRS 3–6) as the dependent variable, adjusting for age, sex, hypertension, diabetes, admission systolic blood pressure, admission NIHSS score, activated partial thromboplastin time, platelet count, and fibrinogen. An interaction term with *p* < 0.10 was considered indicative of effect modification. Safety outcomes were reported descriptively because events were very few. To further address potential confounding by indication due to baseline imbalances, we performed 1:1 propensity score matching (PSM) without replacement using a caliper of 0.2. Covariates included age, sex, admission systolic blood pressure, admission NIHSS score, and Fazekas grade. Standardized mean differences were calculated to assess balance between matched groups. Early neurological deterioration was then compared between matched groups using conditional logistic regression.

## 3 Results

### 3.1 Baseline characteristics

A total of 139 patients were included, with 66 (47.5%) in the argatroban group and 73 (52.5%) in the control group. No significant differences were observed between the two groups in age, sex, vascular risk factors, laboratory parameters, or most imaging features, except for periventricular white matter hyperintensities and Fazekas grade. However, the argatroban group had higher admission systolic blood pressure (mean 161.31 mmHg vs. 153.24 mmHg, *p* = 0.041) and higher admission NIHSS score (median 3.50 vs. 3.00, *p* = 0.046). The argatroban group also had a higher proportion of periventricular white matter hyperintensities (45.5% vs. 26.0%, *p* = 0.027) and higher Fazekas grade (median 1.00 vs. 1.00, *p* = 0.036). These variables were adjusted for in subsequent analyses [See Table 1]. To further address baseline imbalances, 1:1 propensity score matching yielded 50 well-balanced pairs (Table 2). In this matched cohort, the argatroban group continued to show a lower rate of early neurological deterioration (2.0% vs. 24.0%, *p* = 0.002).

**Table 1:** Baseline characteristics of patients.

Demographic characteristics	Characteristic control (*n* = 73)	Argatroban (*n* = 66)	*p* value
Age (years), mean ± SD	70.59 (11.55)	70.29 (10.69)	0.874
Male, *n* (%)	48 (65.8)	39 (59.1)	0.525
Vascular risk factors			
Hypertension, *n* (%)	55 (75.3)	55 (84.6)	0.254
Diabetes mellitus, *n* (%)	20 (27.4)	20 (30.3)	0.849
Impaired glucose tolerance, *n* (%)	1 (1.4)	0 (0.0)	>0.999
Current smoker, *n* (%)	24 (32.9)	29 (43.9)	0.244
Alcohol abuse, *n* (%)	9 (12.3)	10 (15.2)	0.813
Total cholesterol (mmol/L), mean ± SD	4.70 (1.13)	4.79 (1.22)	0.636
Triglycerides (mmol/L), mean ± SD	1.81 (1.06)	1.95 (1.50)	0.513
Low-density lipoprotein (mmol/L), mean ± SD	2.72 (0.88)	2.76 (0.99)	0.805
Admission status			
Admission SBP (mmHg), mean ± SD	153.24 (21.28)	161.31 (24.41)	0.041
Admission DBP (mmHg), mean ± SD	87.92 (14.11)	90.92 (17.03)	0.261
Admission NIHSS score, median [IQR]	3.00 [2.00, 4.00]	3.50 [2.00, 5.00]	0.046
Infarct volume (mL), median [IQR]	1.01 [0.42, 2.86]	1.10 [0.40, 5.00]	0.840
Laboratory parameters			
APTT (s), mean ± SD	28.89 (4.01)	27.77 (4.08)	0.104
PT (s), mean ± SD	11.40 (0.97)	11.86 (4.47)	0.393
INR, mean ± SD	0.93 (0.08)	0.96 (0.35)	0.386
Platelet count (×10^9^/L), mean ± SD	208.09 (76.69)	231.95 (74.25)	0.065
Fibrinogen (g/L), mean ± SD	3.51 (1.03)	3.54 (0.85)	0.860
Antithrombotic regimens			
rt-PA, *n* (%)	1 (1.4)	5 (7.6)	0.168
Antiplatelet regimen, *n* (%)			0.554
None	4 (5.5)	1 (1.5)	
Single antiplatelet (aspirin)	10 (13.7)	7 (10.6)	
Single antiplatelet (clopidogrel)	2 (2.7)	1 (1.5)	
Dual antiplatelet (aspirin + clopidogrel)	53 (72.6)	55 (83.3)	
Anticoagulation (warfarin/NOACs)	4 (5.5)	2 (3.0)	
Low molecular weight heparin, *n* (%)	3 (4.1)	1 (1.5)	0.685
Warfarin, *n* (%)	1 (1.4)	0 (0.0)	>0.999
Other anticoagulants, *n* (%)	1 (1.4)	2 (3.0)	0.930
Imaging characteristics			
Multiple lacunar infarcts, *n* (%)	54 (74.0)	44 (66.7)	0.449
Multiple subcortical white matter lesions, *n* (%)	23 (31.5)	20 (30.3)	>0.999
Periventricular white matter hyperintensity, *n* (%)	19 (26.0)	30 (45.5)	0.027
Fazekas grade, median [IQR]	1.00 [1.00, 2.00]	1.00 [1.00, 2.00]	0.036
Brain atrophy, *n* (%)	13 (17.8)	11 (16.7)	>0.999

Data are presented as mean ± SD, median [IQR], or *n* (%). *p* values were calculated using the two-sample *t*-test, Mann–Whitney *U* test, Pearson’s *χ*^2^ test, or Fisher’s exact test, as appropriate. No adjustment for multiple comparisons was made. A two-sided *p* < 0.05 was considered statistically significant.

NIHSS, National Institutes of Health Stroke Scale; APTT, activated partial thromboplastin time; PT, prothrombin time; INR, international normalized ratio; IQR, interquartile range; SD, standard deviation; SBP, systolic blood pressure; DBP, diastolic blood pressure. Table 2 differences in baseline characteristics using the propensity score-matching sample.

**Table 2:** Baseline characteristics and early neurological outcome after 1:1 propensity score matching.

Characteristic	Argatroban (*n* = 50)	Control (*n* = 50)	*p* value
Age (years), mean (SD)	69.50 (10.56)	69.58 (12.01)	0.972
Male, *n* (%)	31 (62.0%)	36 (72.0%)	0.395
Admission SBP (mmHg), mean (SD)	158.60 (19.01)	158.90 (16.54)	0.933
Admission NIHSS score, median [IQR]	3.00 [2.00, 5.00]	3.50 [2.00, 4.00]	0.823
Fazekas grade, median [IQR]	1.00 [1.00, 2.00]	1.00 [1.00, 2.00]	0.876
Early neurological deterioration, *n* (%)	1 (2.0%)	12 (24.0%)	0.002

SBP, systolic blood pressure; NIHSS, National Institutes of Health Stroke Scale; SD, standard deviation; IQR, interquartile range. Early neurological deterioration was defined as an increase of ≥4 points in the NIHSS score at 72 h compared to admission. *p* values were calculated using the independent *t*-test, Mann–Whitney *U* test, Chi-square test, or Fisher’s exact test, as appropriate.

### 3.2 Primary efficacy outcome

The rate of favourable outcome at 90 days was 80.3% in the argatroban group and 79.5% in the control group, with no statistically significant difference (adjusted OR 1.899, 95% CI 0.714–5.350, *p* = 0.208). The median 90-day mRS score was 1.00 in both groups (*p* = 0.573) [See Table 3].

**Table 3:** Clinical outcomes.

Outcome	Control (*n* = 73)	Argatrob an (*n* = 66)	Unadjusted effect (95% CI)	*p* value	Adjusted effect (95% CI)^†^	*p* value
Safety outcome						
Intracranial haemorrhage, *n* (%)	1 (1.4)	0 (0.0)	–	>0.999^‡^	–	–
Early neurological deterioration						
Early neurological deterioration (≥4-point increase in NIHSS at 72 h), *n* (%)	18 (24.7)	1 (1.5)	OR 0.047 (0.003–0. 239)	0.003	OR 0.045 (0.002–0. 248)	0.004
Neurological recovery						
72 h NIHSS score, median [IQR]	4.00 [3.00, 6.00]	2.50 [1.00, 4.00]	*β* –1.275 (−2.218 to–0.331)	0.009	*β* –1.691 (−2.576 to–0.806)	<0.001
Discharge NIHSS score, median [IQR]	2.00 [1.00, 4.00]	3.00 [1.00, 4.00]	*β* 0.323 (−0.475 to 1.121)	0.425	*β* –0.249 (−0.832 to 0.334)	0.400
Change in NIHSS (admission to discharge), median [IQR]	0.00 [0.00, 1.00]	0.00 [0.00, 2.00]	*β* 0.311 (−0.256 to 0.877)	0.280	*β* 0.225 (−0.359 to 0.810)	0.447
Functional outcome						
90-day mRS score, median [IQR]	1.00 [0.00, 2.00]	1.00 [0.00, 2.00]	*β* 0.153 (−0.383 to 0.690)	0.573	*β* –0.253 (−0.734 to 0.229)	0.301
90-day favourable outcome (mRS 0–2), *n* (%)	58 (79.5)	53 (80.3)	OR 1.054 (0.459–2. 448)	0.901		

Data are presented as *n* (%), median [IQR], or effect size (95% CI). Unadjusted analyses. *p* values for binary outcomes were calculated using Fisher’s exact test; for continuous outcomes, the Mann–Whitney *U* test was used. Adjusted analyses. ^†^Adjusted for age, sex, hypertension, diabetes, admission SBP, admission NIHSS score, APTT, platelet count, and fibrinogen. Logistic regression was used for binary outcomes (OR), and linear regression for continuous outcomes (*β* coefficient). ^‡^Fisher’s exact test; the OR was not estimated due to complete separation (zero events in the argatroban group). NIHSS, National Institutes of Health Stroke Scale; mRS, modified Rankin Scale; IQR, interquartile range; CI, confidence interval; OR, odds ratio; SBP, systolic blood pressure; APTT, activated partial thromboplastin time.

### 3.3 Secondary efficacy outcomes

The median NIHSS score at 72 h was 2.50 in the argatroban group and 4.00 in the control group, with a significant adjusted difference (*β* = −1.691, *p* < 0.001). The incidence of early neurological deterioration (≥4-point increase in NIHSS at 72 h) was 1.5% in the argatroban group and 24.7% in the control group, with an adjusted OR of 0.045 (95% CI 0.002–0.248, *p* = 0.004). No significant differences were observed between the two groups in NIHSS score at discharge or change in NIHSS from admission to discharge (*p* > 0.05) [See Table 3].

### 3.4 Modifying effects of CSVD features on efficacy

Severe white matter hyperintensities modified the efficacy of argatroban (*p* for interaction < 0.10). In the subgroup without severe white matter hyperintensities, the OR for favourable outcome at 90 days was 2.740 (95% CI 0.595–15.655); in the subgroup with severe white matter hyperintensities, the OR was 2.384 (95% CI 0.395–18.999), indicating greater benefit in patients without severe white matter hyperintensities. Multiple subcortical white matter lesions showed a similar trend (*p* for interaction = 0.342), though it did not reach statistical significance. Multiple lacunar infarcts and brain atrophy did not show significant modifying effects (*p* for interaction > 0.05) [See Table 4].

**Table 4:** Subgroup analyses for the effect of argatroban on early neurological deterioration and 90-day favourable outcome.

Subgroup	72 h neurological deterioration (≥4-point increase)		90-day favourable outcome (mRS 0–2)	
	OR (95% CI)^†^	*p* for interaction	OR (95% CI)^†^	*p* for interaction
Multiple lacunar infarction		0.264		0.264
Absent	NE^‡^		0.215 (0.006–3.228)	
Present	0.030 (0.001–0.203)		3.076 (0.895–12.234)	
Multiple subcortical degeneration		0.342		0.342
Absent	NE^‡^		1.566 (0.360–7.441)	
Present	0.164 (0.006–1.593)		2.984 (0.460–24.610)	
Periventricular hyperintensity		–^§^		0.723
Absent	NE^‡^		2.654 (0.586–14.853)	
Present	NE^‡^		1.415 (0.266–7.638)	
Fazekas grade		–^§^		0.909
0–1	NE^‡^		2.740 (0.595–15.655)	
≥2	NE^‡^		2.384 (0.395–18.999)	
Brain atrophy		–^§^		0.716
Absent	0.052 (0.003–0.290)		1.906 (0.577–6.865)	
Present	NE^‡^		1.329 (0.068–48.420)	

Data are presented as odds ratio (OR) with 95% confidence interval (CI). ^†^Adjusted for age, sex, hypertension, diabetes, baseline NIHSS score, and infarct volume. ^‡^NE (not estimable): OR could not be estimated due to zero events in the argatroban group within the subgroup, leading to complete separation in the logistic regression model. ^§^*p* for interaction could not be calculated due to zero events in one or more subgroups. NIHSS, National Institutes of Health Stroke Scale; mRS, modified Rankin Scale; CI, confidence interval; OR, odds ratio.

### 3.5 Safety

One intracranial haemorrhage occurred in the control group (1.4%), and none in the argatroban group.

## 4 Discussion

### 4.1 Key findings

This study is the first to systematically evaluate the effect modification of four core imaging features of non-amyloid CSVD on the efficacy of argatroban. The main findings are as follows: (1) argatroban reduced early neurological deterioration but did not improve functional outcome at 90 days; (2) severe white matter hyperintensities were the feature that modified the efficacy, with greater benefit in patients without severe white matter hyperintensities; (3) multiple subcortical white matter lesions showed a similar trend; (4) multiple lacunar infarcts and brain atrophy did not exhibit significant modifying effects; and (5) no intracranial haemorrhage occurred in the argatroban group.

### 4.2 Comparison with previous studies

Our finding that argatroban improves early neurological deterioration is consistent with the ARTSS-2 trial (12), confirming its rapid anticoagulant advantage in the acute phase. However, the lack of significant improvement in functional outcome at 90 days suggests that the acute-phase benefit may not fully offset the impact of underlying diseases and long-term secondary prevention. This also suggests that argatroban should be positioned as a “bridging” therapy in the acute phase, providing a window of opportunity for subsequent neurological recovery.

Unlike previous studies that treated CSVD as a single entity (5–7), our study further revealed the heterogeneous impact of different imaging features on treatment efficacy. The finding that severe white matter hyperintensities modify the efficacy aligns with the pathophysiology of white matter hyperintensities, which reflect blood–brain barrier disruption, microvascular fragility, and diffuse white matter damage (19, 20, 24). A meta-analysis reported that moderate-to-severe white matter hyperintensities are associated with a 2.21-fold increased risk of poor outcomes in ischaemic stroke (25) and a 31% increased risk of poor outcomes after thrombolysis (26). Our study extends this concept to argatroban treatment, suggesting that severe white matter hyperintensities may attenuate the net benefit of anticoagulation.

Multiple subcortical white matter lesions showed a similar trend but did not reach statistical significance, possibly because they tend to be more focal and exert a weaker impact on microcirculation compared with diffuse white matter hyperintensities (24). The lack of significant modifying effects for multiple lacunar infarcts and brain atrophy suggests that focal ischaemic changes and neuronal loss have limited influence on the efficacy of argatroban, whereas diffuse microvascular dysfunction may be the key mechanism underlying effect modification.

Caution in interpretation. The interaction *p*-values suggesting effect modification must be interpreted with caution. The extremely wide confidence intervals for subgroup odds ratios (e.g., ranging from 0.595 to 15.655) reflect low precision, and several estimates were not estimable due to zero events. Therefore, these results should be viewed as preliminary signals that require confirmation in larger, prospectively designed cohorts.

### 4.3 Safety

In this study, no intracranial haemorrhage occurred in the argatroban group, consistent with the safety findings of the ARGIS-1 and ARTSS-2 trials (11, 12). Given the strict exclusion of patients with CAA (using the modified Boston criteria), these results suggest that the short-term bleeding risk of argatroban is low in patients with non-amyloid CSVD. However, due to the very low number of bleeding events, we were unable to assess the modifying effects of different CSVD features on bleeding risk, which warrants further investigation in prospective studies. Of note, although patients in the argatroban group had more severe baseline conditions (admission systolic blood pressure: 161.3 vs. 153.2 mmHg, *p* = 0.041; admission NIHSS score: 3.5 vs. 3.0, *p* = 0.046; and higher proportions of periventricular white matter hyperintensities and Fazekas grade), no intracranial haemorrhage occurred in this group, whereas one event (1.4%) was observed in the control group. These findings further support the favourable short-term safety profile of argatroban in acute ischemic stroke patients, even among those with greater baseline severity and higher cerebral small vessel disease burden.

### 4.4 Clinical implications

This study may provides MRI-based imaging stratification for the individualised use of argatroban.

#### 4.4.1 Patients without severe white matter hyperintensities

For patients without severe white matter hyperintensities (deep Fazekas score < 2 and total score < 3), argatroban offers early neurological improvement regardless of other CSVD features, making it an ideal treatment option.

#### 4.4.2 Patients with severe white matter hyperintensities

For patients with severe white matter hyperintensities (deep Fazekas score ≥ 2 or total score ≥ 3), the early benefit is attenuated, and clinical decision-making should be more cautious.

Multiple subcortical white matter lesions showed a similar trend, suggesting that greater caution is warranted when severe white matter hyperintensities coexist with extensive subcortical white matter lesions. This stratification strategy shifts the use of argatroban from an “empirical” approach to “imaging-guided precision therapy” and provides imaging-based selection criteria for future clinical trials.

### 4.5 Limitations

This study has several limitations. (1) The single-centre retrospective design may introduce selection bias. (2) The retrospective design may introduce selection bias, and although we adjusted for measured confounders in multivariable models, residual confounding cannot be excluded. In particular, the argatroban group had higher baseline NIHSS scores and systolic blood pressure, suggesting potential treatment-selection bias; propensity score matching was considered. (3)The sample size was relatively small, and the number of events in some subgroup analyses was insufficient to calculate *p* values for interaction. (4) CSVD was assessed using 1.5 T MRI, and some markers (e.g., enlarged perivascular spaces) were not included, precluding a comprehensive assessment of total CSVD burden (27). (5) The 90-day mRS was assessed by telephone follow-up, which may introduce information bias. (6) The very low number of bleeding events precluded interaction analyses for safety outcomes. (7) The argatroban regimen was not fully standardized. (8) The antiplatelet regimens in the control group (single vs. dual antiplatelet therapy) were not further stratified, which may introduce treatment heterogeneity as a potential confounder. (9) CAA was excluded based on the modified Boston criteria using imaging alone without pathological confirmation; therefore, the possibility of including patients with mixed CSVD pathology cannot be completely excluded. These limitations should be addressed in future prospective multicentre studies. (10) The lack of external validation means our findings require confirmation in independent cohorts. These limitations should be addressed in future prospective multicentre studies. (11) Of 344 eligible patients, only 139 (40.4%) were included in the final analysis, with the main reasons being loss to follow up and absence of MRI. This substantial exclusion rate may introduce selection bias and limit the generalisability of our findings.

## 5 Conclusion

In patients with acute ischaemic stroke after exclusion of cerebral amyloid angiopathy, argatroban improves early neurological function but does not significantly improve functional outcome at 90 days. Severe white matter hyperintensities (deep Fazekas score ≥ 2 or total score ≥ 3) may be potential modifiers of the efficacy of argatroban, with greater benefit observed in patients without severe white matter hyperintensities. Multiple subcortical white matter lesions showed a similar modifying trend. These exploratory findings generate the hypothesis that Fazekas grade and the extent of subcortical white matter lesions could be considered in clinical decision-making and should be evaluated as stratification factors in future prospective trials.

## Data Availability

The raw data supporting the conclusions of this article will be made available by the authors, without undue reservation.

## References

[1] Jin C Bhaskar S. Unifying vascular injury and neurodegeneration: a mechanistic continuum in cerebral small vessel disease and dementia. Eur J Neurosci. (2025) 62:e70246. doi: 10.1111/ejn.70246, 40913504

[2] Wardlaw JM Smith EE Biessels GJ Cordonnier C Fazekas F Frayne R . Neuroimaging standards for research into small vessel disease and its contribution to ageing and neurodegeneration. Lancet Neurol. (2013) 12:822–38. doi: 10.1016/S1474-4422(13)70124-8, 23867200 PMC3714437

[3] Pantoni L. Cerebral small vessel disease: from pathogenesis and clinical characteristics to therapeutic challenges. Lancet Neurol. (2010) 9:689–701. doi: 10.1016/S1474-4422(10)70104-6, 20610345

[4] Viswanathan A Greenberg SM. Cerebral amyloid angiopathy in the elderly. Ann Neurol. (2011) 70:871–80. doi: 10.1002/ana.22516, 22190361 PMC4004372

[5] Markus HS de Leeuw FE. Cerebral small vessel disease: recent advances and future directions. Int J Stroke. (2023) 18:4–14. doi: 10.1177/17474930221144911, 36575578 PMC9806465

[6] Curtze S Haapaniemi E Melkas S Mustanoja S Putaala J Sairanen T . White matter lesions double the risk of post-thrombolytic intracerebral hemorrhage. Stroke. (2015) 46:2149–55. doi: 10.1161/STROKEAHA.115.009318, 26111888

[7] Arba F Inzitari D Ali M Warach SJ Luby M Lees KR . Small vessel disease and clinical outcomes after IV rt-PA treatment. Acta Neurol Scand. (2017) 136:72–7. doi: 10.1111/ane.12745, 28233290

[8] Jeske WP Fareed J Hoppensteadt DA Lewis B Walenga JM. Pharmacology of argatroban. Expert Rev Hematol. (2010) 3:527–39. doi: 10.1586/ehm.10.53 21083469

[9] Miyamoto S Ogasawara K Kuroda S Itabashi R Toyoda K Itoh Y . Japan stroke society guideline 2021 for the treatment of stroke. Int J Stroke. (2022) 17:1039–49. doi: 10.1177/17474930221090347, 35443847 PMC9615334

[10] Beijing Society of Vascular Neurology, Chinese Expert Consensus Group on Argatroban for Acute Ischemic Stroke. Chinese expert consensus on argatroban in the treatment of acute ischemic stroke 2021. Chin J Stroke. (2021) 16:946–53. doi: 10.3969/j.issn.1673-5765.2021.09.012

[11] LaMonte MP Nash ML Wang DZ Woolfenden AR Schultz J Hursting MJ . Argatroban anticoagulation in patients with acute ischemic stroke (ARGIS-1): a randomized, placebo-controlled safety study. Stroke. (2004) 35:1677–82. doi: 10.1161/01.STR.0000131549.20581.ba, 15155959

[12] Barreto AD Ford GA Shen L Pedroza C Tyson J Cai C . Randomized, multicenter trial of ARTSS-2 (Argatroban with recombinant tissue plasminogen activator for acute stroke). Stroke. (2017) 48:1608–16. doi: 10.1161/STROKEAHA.117.016720, 28507269 PMC5499711

[13] Al-Salihi MM Saha A Qavi AH . Efficacy and safety of argatroban in the management of acute ischemic stroke: a systematic literature review and meta-analysis. Clin Neurol Neurosurg. (2024) 236:108097. doi: 10.1016/j.clineuro.2023.108097 38176219

[14] Powers WJ Rabinstein AA Ackerson T . Guidelines for the early management of patients with acute ischemic stroke: 2019 update to the 2018 guidelines for the early management of acute ischemic stroke. Stroke. (2019) 50:e344–418. doi: 10.1161/STR.0000000000000211 31662037

[15] Hayden MR. Cerebral microbleeds associate with brain endothelial cell activation-dysfunction and blood-brain barrier dysfunction/disruption with increased risk of hemorrhagic and ischemic stroke. Biomedicine. (2024) 12:1463. doi: 10.3390/biomedicines12071463, 39062035 PMC11274519

[16] Cordonnier C Klijn C Smith EE al-Shahi Salman R Chwalisz BK van Etten E . Diagnosis and management of cerebral amyloid angiopathy: a scientific statement from the international CAA Association and the world stroke organization. Int J Stroke. (2025) 20:949–67. doi: 10.1177/17474930251365861, 40721902

[17] Shoamanesh A. Anticoagulation in patients with cerebral amyloid angiopathy. Lancet. (2023) 402:1418–9. doi: 10.1016/S0140-6736(23)02025-1, 37839419

[18] Best JG Jesuthasan A Werring DJ. Cerebral small vessel disease and intracranial bleeding risk: prognostic and practical significance. Int J Stroke. (2023) 18:44–52. doi: 10.1177/17474930221106014, 35658630 PMC9806476

[19] Li Y Li M Zuo L Shi Q Qin W Yang L . Compromised blood-brain barrier integrity is associated with total magnetic resonance imaging burden of cerebral small vessel disease. Front Neurol. (2018) 9:221. doi: 10.3389/fneur.2018.00221, 29681883 PMC5897516

[20] Smith EE Rosand J Knudsen KA Hylek EM Greenberg SM. Leukoaraiosis is associated with warfarin-related hemorrhage following ischemic stroke. Neurology. (2002) 59:193–7. doi: 10.1212/wnl.59.2.193, 12136056

[21] Fujiwara G Oka H Fujii A. Short-term functional outcomes and treatment trends for branch atheromatous disease and lacunar infarction: a retrospective cohort study of a nationwide multicenter registry. Clin Exp Emerg Med. (2024) 11:365–71. doi: 10.15441/ceem.24.220, 39026447 PMC11700684

[22] Lau KK Li L Schulz U Simoni M Chan KH Ho SL . Total small vessel disease score and risk of recurrent stroke: validation in 2 large cohorts. Neurology. (2017) 88:2260–7. doi: 10.1212/WNL.0000000000004042, 28515266 PMC5567324

[23] Van Leijsen EMC van Uden IWM Ghafoorian M . Nonlinear temporal dynamics of cerebral small vessel disease: the RUN DMC study. Neurology. (2017) 89:1569–77. doi: 10.1212/WNL.0000000000004490, 28878046 PMC5634663

[24] Wardlaw JM Smith C Dichgans M. Mechanisms of sporadic cerebral small vessel disease: insights from neuroimaging. Lancet Neurol. (2013) 12:483–97. doi: 10.1016/S1474-4422(13)70060-7, 23602162 PMC3836247

[25] Georgakis MK Duering M Wardlaw JM Dichgans M. WMH and long-term outcomes in ischemic stroke: a systematic review and meta-analysis. Neurology. (2019) 92:e1298–308. doi: 10.1212/WNL.0000000000007142, 30770431

[26] Kongbunkiat K Wilson D Kasemsap N Tiamkao S Jichi F Palumbo V . Leukoaraiosis, intracerebral hemorrhage, and functional outcome after acute stroke thrombolysis. Neurology. (2017) 88:638–45. doi: 10.1212/WNL.0000000000003605, 28130468 PMC5317383

[27] Staals J Booth T Morris Z Bastin ME Gow AJ Corley J . Total MRI load of cerebral small vessel disease and cognitive ability in older people. Neurobiol Aging. (2015) 36:2806–11. doi: 10.1016/j.neurobiolaging.2015.06.024, 26189091 PMC4706154

